# Education and support needs during recovery in acute respiratory distress syndrome survivors

**DOI:** 10.1186/cc8053

**Published:** 2009-09-23

**Authors:** Christie M Lee, Margaret S Herridge, Andrea Matte, Jill I Cameron

**Affiliations:** 1 Division of Respirology, University of Toronto, 1 King's College Circle, 6263 Medical Sciences Building, Toronto, ON, M5S 1A8, Canada; 2 Interdepartmental Division of Critical Care, University Health Network, University of Toronto, 399 Bathurst Street, Toronto, ON, M5T 2S8, Canada; 3 Department of Occupational Science and Occupational Therapy, University of Toronto, 160 - 500 University Avenue, Toronto, ON, M5G 1V7, Canada; 4 Toronto Rehabilitation Institute, 550 University Avenue, Toronto, ON, M5G, Canada

## Abstract

**Introduction:**

There is a limited understanding of the long-term needs of survivors of the acute respiratory distress syndrome (ARDS) as they recover from their episode of critical illness. The Timing it Right (TIR) framework, which emphasizes ARDS survivors' journey from the ICU through to community re-integration, may provide a valuable construct to explore the support needs of ARDS survivors during their recovery.

**Methods:**

Twenty-five ARDS survivors participated in qualitative interviews examining their needs for educational, emotional and tangible support for each phase of the TIR framework. Transcripts were analyzed using framework methodology.

**Results:**

ARDS survivors' support needs varied across the illness trajectory. During the ICU stay, survivors were generally too ill to require information. The transfer to the general ward was characterized by anxiety surrounding decreased surveillance and concern for future health and treatment. Information needs focused on the events surrounding the acute illness, while physical and emotional needs revolved around physical therapy and psychological support for depression and anxiety. As patients were preparing for hospital discharge, they expressed a desire for specific information about the recovery and rehabilitation process following an episode of ARDS (e.g., outpatient physiotherapy, long-term sequela of the illness). Once in the community, survivors wanted guidance on home care, secondary prevention, and ARDS support groups.

**Conclusions:**

Our findings support the need for future educational and support interventions to meet the changing needs of ARDS survivors during their recovery.

## Introduction

Acute respiratory distress syndrome (ARDS) is an important public health concern with an incidence of 1.5 to 8.3 cases per 100,000 per year in North America [1-3]. It is characterized by bilateral lung infiltrates on frontal chest radiograph, a partial pressure of arterial oxygen (PaO_2_)/fraction of inspired oxygen (FiO_2_) ratio of 200 or less, and the absence of clinical evidence of left atrial hypertension [4]. Survivors experience physical disability in the form of muscle wasting and weakness, and diminished ability to exercise up to five years after discharge from the ICU [5-7]. In addition, they also sustain important neuropsychological issues including depression, anxiety, memory loss, and difficulty with concentration [6,8-19]. Fewer than half of all ARDS survivors return to work within the first year following ICU discharge [5], two-thirds return to the work force at two years [6], and 77% of all ARDS survivors return to work at five years [7]. Return to work has been shown to be inversely related to the severity of depression experienced by ARDS survivors [20], but despite this, most survivors continue to report functional limitation measured as distance walked in six minutes and a reduction in their physical quality of life.

A small body of research has begun to explore the support needs of ICU survivors. During the acute phase of critical illness, informational needs include: nature of illness/treatments; prognosis; impact of treatment; potential complications; and expected care needs after hospitalization [21]. Patients and families also report fragmentation of care associated with transfers between the ICU and the general ward [22] and between acute care and the community [23]. Much less is known about the support needs of ARDS survivors during inpatient rehabilitation or during the first months to years back in the community.

In the recent stroke literature, the 'Timing it Right' (TIR) framework has provided a construct in which to examine the changing needs of stroke caregivers from acute care, through rehabilitation, and back to community living [24]. The TIR framework is the first of its kind to try and articulate how experiences and support needs evolve from an acute illness through recovery and community re-integration. Although it was developed specifically for family caregivers to stroke survivors, the central premise - that support needs evolve over time - applies to patient populations as well as illness populations other than stroke. This framework can be used to identify phase-specific needs to inform programs to enhance readiness and ease transitions across care environments.

The framework consists of five-distinct phases that consider the timing, setting, focus of care, support needs, and modifiable outcomes for each phase. Our clinical research team has adapted the TIR framework to reflect the dominant phases of recovery for ICU survivors. The adapted five phases include: 1) the critical illness event and ICU care; 2) period of stabilization on the general ward; 3) preparation for return to community living; 4) first few months of home adjustment, and 5) longer-term adjustment to community living [24]. The framework emphasizes four aspects of support: informational, emotional, instrumental (e.g., tangible assistance from health care professionals, training to self-care), and feedback from others about how they are managing [25]

Currently, in the USA, formal follow-up for survivors of critical illness is not a part of standard clinical practice. In contrast, approximately one-third of all hospitals in the UK provide some form of post-ICU and follow-up care [26]. Until recently, much of this care was led by nursing staff and because no formal guidelines existed, care was variable across the region [26]. In 2009, the National Institute for Health and Clinical Excellence developed a consensus statement to address the care of patients following a period of critical illness [27]. The guidelines address many aspects of post-ICU care including information, support and rehabilitation. [27]. Despite these new guidelines, at present, there is limited data to inform programs to support the long-term recovery of this population. This study uses the TIR framework to explore the support needs of ARDS survivors during and after their acute episode of critical illness as they re-integrate into the community.

## Materials and methods

### Design

We conducted a qualitative study using in-depth interviews and framework methodology [28,29] to guide data collection and analysis. The qualitative design ensures a rich description of individuals' experiences and needs [30] and may be informative when developing complex rehabilitative and educational interventions [31].

### Participants

Participants were recruited from the Toronto ARDS survivor cohort and followed through November 2006 [5]. This study involved clinical follow-up at three months after ICU discharge, then every six months for a total of five years. Inclusion and exclusion criteria were cited in detail previously [5]. Briefly, patients were eligible for enrollment into the cohort study if they were at least 16 years of age, with a PaO_2_/FiO_2 _ratio of 200 or less while receiving mechanical ventilation with a positive end-expiratory pressure of at least 5 cmH_2_O, evidence of air-space changes in all four quadrants on chest radiography, and an identifiable risk factor for the ARDS. Patients were excluded if they were immobile before being admitted to the ICU, had a history of pulmonary resection, or had a documented neurologic or psychiatric disease. At the five-year follow-up, 64 patients remained in the cohort study. All surviving patients were mailed a study information package, a study description, and an invitation to participate in this qualitative study. Consent forms could be returned by mail or via fax. One week after sending these packages, one member of the research team contacted each participant via telephone to provide them with a full description of the study and answer any questions. All interested participants were then asked to arrange a time with one of two members of the research team, (CL or AM) to conduct the interview. Participation in this study was on a voluntary basis. Interviews were conducted until theme saturation was achieved.

### Data collection

In-depth qualitative interviews followed a structured interview guide to focus the discussion on participants' experiences and needs for informational, instrumental, and emotional support from their ICU experience through their community re-integration and corresponding to the phases of the TIR Framework (see interview guide in Table 1). The interview asked a series of questions for each of five phases that we adapted from the TIR framework: 1) the critical illness event and ICU care; 2) period of stabilization on the general ward; 3) preparation for return to community living; 4) first few months of home adjustment; and 5) longer-term adjustment to community living. For each phase, interviewers asked participants to indicate what support they did receive, how and from whom they received it, and what support they would have liked but did not receive. In situations where needs were not being met, participants were invited to suggest how these needs could be met and by whom.

**Table 1:** ARDS survivor interview guide


1. Please describe your experience with ARDS beginning in the ICU, moving through acute care and rehab, and then back to the community.
2. Do you currently have any physical, emotional, cognitive difficulties?
3. Please describe your ICU stay to me. Elaborate on any met or unmet needs that you may have experienced. What could have made your stay easier or more comfortable? Were there any features during your stay that troubled you?
4. Please describe your ward stay for me. Elaborate on any met or unmet needs that you may have experienced. How were these needs different from your ICU stay? Were you concerned about leaving the ICU?
5. Please describe your experiences as you prepared for hospital discharge. Elaborate on any met or unmet needs that you may have experienced. What was your destination following discharge - rehabilitation hospital or home?
6. Please tell me about your experiences during in-patient rehabilitation. Elaborate on any met or unmet needs that you may have experienced.
7. Please tell me about your experiences during your first few months at home. Elaborate on any met or unmet needs that you may have experienced.
8. Please tell me about your experiences living back in the community. How were they different from your first few months? Elaborate on any met or unmet needs that you may have experienced.
9. What are your thoughts regarding your future? Do you foresee any further support needs? Please elaborate on any needs you think you may require.
10. As you reflect back on your entire illness, what specific times in your recovery would you have benefited most from physical rehabilitation, psychological counseling, information, education, support, and/or training?

ARDS = acute respiratory distress syndrome;

All interviews were conducted either in-person or by telephone because the quantity and quality of data colected in these two ways have been shown to be comparable [32]. Interviews were 45 to 90 minutes in duration, audio taped, and professionally transcribed verbatim. Each participant was interviewed once during the study.

### Data analysis

Evaluation of the in-depth interviews followed the five stages of framework analysis: 1) familiarizing by listening to the interviews and reviewing the transcripts; 2) selecting a thematic framework (e.g. TIR model); 3) coding the data according to the framework; 4) charting the data on the framework, and 5) interpretation [28,29]. This approach allowed us to outline changing education, support, and rehabilitation needs across the phases of the TIR framework and to identify any non-phase-based themes. To minimize the threat of bias entering the analysis we used the following strategies as recommended by McReynolds and colleagues [33]: we maintained an audit trail by keeping record of all data analysis procedures and notes; multiple researchers contributed to the data analysis and theme generation; and we examined discrepant data [33]. Specifically, two additional authors (JC and MH) contributed to data analysis. We used NVivo qualitative software (QSR International, Cambridge, MA, USA) to organize the coding process.

### Ethics approval

This study was approved under the University Health Network Research Ethics Board, reference number 06-0164AE.

## Results

### Participants

Twenty-five ARDS survivors participated in the study. Interviews varied between 45 and 90 minutes in length. Thematic saturation was achieved through convenience sampling. The characteristics of the study participants are summarized in Table 2. Thirty-nine individuals declined participation for various reasons. These reasons are summarized in Table 3.

**Table 2:** Baseline characteristics of ARDS survivor in qualitative study compared with cohort at five years

Baseline characteristics	Qualitative study n = 25 (%)	Non-participants n = 39 (%)	ARDS cohort at five years n = 64 (%)
Proportion of females	11 (45.8)	18 (46)	31 (48)
Age (years)*	48.3	40.5	44
Proportion with spouse	18 (75)	-	-
Time since ICU discharge (months)*	75.5	77.9	79
APACHE II score¶*	24	21	23
Maximal lung injury score§*	4	3.67	3.7
Length of stay in ICU (days)*	28	25	26
Rehabilitation:			
Inpatient	4 (16)	11(29)	14 (22)
Outpatient	19 (76)	26(67)	44 (69)
Education (post-secondary level)	16 (66.7)	21(72.4)	52 (81.2)
Annual family income ($CDN)*	40,000-49,000	40,000-49,000	40,000-49,000

*Median values

¶APACHE = Acute Physiology, Age, and Chronic Health Evaluation. Scores can range from 0 to 71; higher scores indicate more severe illness.

§Static compliance was not measured. The cumulative score was the sum of the chest x-ray, hypoxemia and positive end expiratory pressure scores. Scores can range from 0 to 4; higher scores indicate more severe lung injury.

ARDS = acute respiratory distress syndrome; $CDN = currency in Canadian dollars.

**Table 3:** Summary table describing reasons for non-participation

Reasons for non-participation	Number of non-participants n = 39 (%)
Inability to obtain a translator	2 (5)
Medically unwell/in hospital	1 (2.5)
Deceased at time of contact	4 (10)
Not interested in participation	9 (23.5)
Failure to respond despite three repeated attempts to contact by telephone	23 (59)

### Overall themes

**Figure 1:**
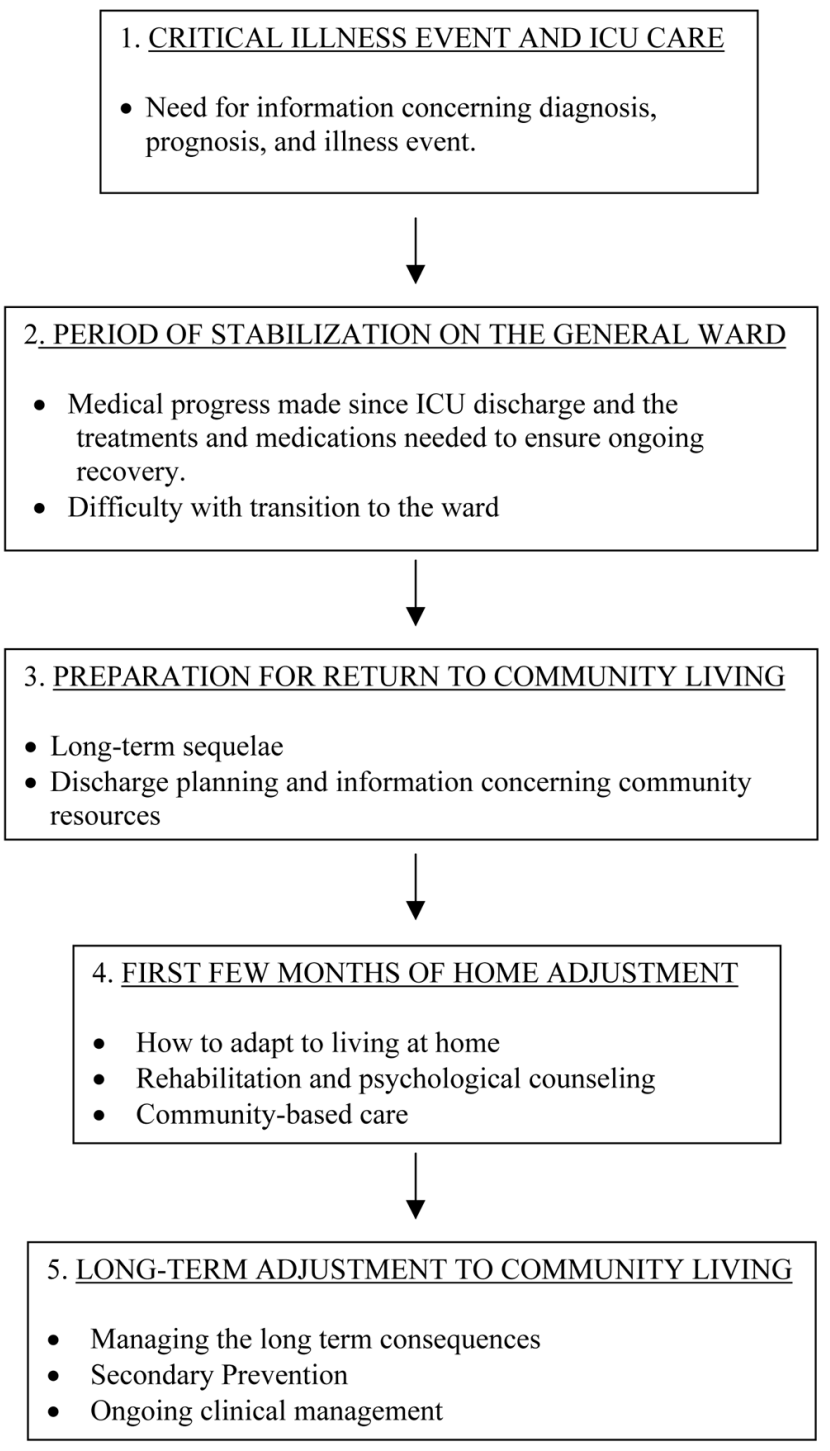
Key characteristics of survivors' needs and experiences by phase of recovery. Flow chart summarizing the five key phases of recovery in acute respiratory distress syndrome (ARDS) survivors from ICU admission to long-term re-integration within the community. Key support needs were identified within each of the five phases of recovery. This flowchart also emphasizes that support needs evolve over time and needs that are not addressed early on in the recovery process can persist through subsequent phases.

The dominant themes identified in each of the five phases are: information needs and emotional support in the critical illness phase; physical rehabilitation and psychological counseling during the stabilization phase on the general ward; expectations for recovery and availability of community services during the preparation for discharge phase; support surrounding adaptation to independent living and overcoming emotional abandonment dominated in the early home adjustment phase; and finally support needs geared towards secondary prevention of health events, health maintenance, and re-integration into society were the main supports identified in the long-term adjustment phase. It is also important to note that certain needs persisted through each of the phases, in particular, that family caregivers were an important source of support for ARDS survivors, that information should always be provided in understandable language to the patients, and that emotional reactions were mixed in each of the phases, perhaps reflecting the variability associated with available support from a family caregiver. Those patients with a caregiver tended to experience more positive emotions in each of the phases. Figure 1 represents a flow chart summary of the key characteristics identified by phase of recovery. In the following sections we will discuss each phase, present the key support needs (i.e., informational, emotional, and tangible supports), describe how each of these needs is unique to each phase, and use quotations from participants as illustrations. In addition, we will discuss how the mechanism for providing and/or receiving support varied across the phases of the TIR framework.

### The critical illness event and ICU care

During the critical illness event and ICU phase many patients were too ill to have specific support needs. A small number of patients reflected on needs centered on the diagnosis, prognosis, and illness event. Family members became a key source of information for the patients. As a result, one key support need during this phase was the importance of transferring knowledge from health care teams to families. One participant discusses the role of family members in receiving information from members of the health care team:

"Information...support...I don't know, they were talking to my family about everything the doctors...it does help that the family get all the information, if the patient can't think...", *ARDS survivor number 118*.

Although family members were viewed as key recipients of information, patients still wanted to be informed about what was happening to them. One participant indicated:

"ICU I was in the dark...I didn't know what the heck was happening to me", *ARDS survivor number 166*.

Participants also highlighted the need to explain things clearly and in a language that patients and family could understand. For example, the following quotation highlights how information could be relayed:

"I, think, if they could have explained things in simpler terms, instead of so many doctor language you know...so that the layman could understand," *ARDS survivor number 227*.

Patients experienced mixed emotions concerning their ICU stay. Many patients felt a sense of fear and anxiety over the complexities of their illness and lack of understanding about their disease. For example, some aspects of the following participant's ICU experience frightened them:

"I remember...being petrified one night....crying to my mother...I couldn't sleep...I was so scared they were going to come and stab me...with needles...one nurse...very nice...said we will not stab you when you're asleep...made me feel better," *ARDS survivor number 363*.

This supports the need for health care professionals to forewarn or prepare the patients before performing procedures or personal care duties. Although some patients experienced fear and anxiety, many also felt a sense of happiness and security because they knew their families were nearby and felt well taken care of by the hospital staff.

"I had the support from the nurses,...my family members,...because they were there, it was comforting to know someone was there...to keep me calm and relaxed too at the same time,"*ARDS survivor number 175*.

In summary, the critical illness event and ICU phase was characterized by the need for care and support concerning the health event. A few patients did highlight the importance of receiving physical therapy while in the ICU, but most had very few recollections in this phase.

### Period of stabilization on the general ward

This phase is characterized by stabilization of the medical condition and transfer to the medical ward. Patients' needs begin to focus on medical progress made since ICU discharge and the treatments and medications needed to ensure ongoing recovery. The information needs continue to focus on the illness event and prognosis, as patients who were previously in a coma were beginning to realize the nature of their own disease. Family members became a key source of support for the patients throughout the recovery process. The survivors relied on their caregivers and family members to fill in gaps in their memory regarding their acute illness. They were also beginning to think about their future health care needs. The following quotation captures this complex time:

"I started to realize what had really happened to me, like the whole self-realization about oh well, I can't quite walk, we needed more support like about, like when I had to go to other places,...we need support so the wheelchair taxis and wheelchair services and more about it...kind of now starting to look beyond the hospital, and ....so it's the needs for support, and information definitely greater, because I was more lucid and we were looking at the future," *ARDS survivor number 352*.

In this phase, transition to the ward was emotionally challenging. Many patients went through a period of intense frustration because of difficulty coping with change in their daily routine. The health care teams were new and the decrease in acuity meant less monitoring and more independent effort from the patients. This participant discussed their experience with the lower level of clinical monitoring:

"It was scarier...on the floor...I was in a room and there was no one else in the room...I felt less people monitoring me...I was more left alone and ...it was more a psychological thing," *ARDS survivor number 419*.

At the same time participants were realizing the extent of their illness and this caused emotional turmoil. For example, this participant felt they were in a "hole" as a result of the physical consequences of their illness and in need of psychological counseling:

"Everyday...I was realizing a little bit more...how deep the hole was...so every step that I actually learned that oh you don't have hair anymore, oh you only weigh 90 pounds, no you're not eating anymore,...you just fall deeper and deeper, into this big hopeless hole of depression...they didn't have a psychiatrist come and meet my needs,...until the middle of October when I was ...extremely depressed," *ARDS survivor number 352*.

On a positive note, when family was available, patients did find comfort from family members who were able to stay in the hospital with them and provide comfort:

"I knew [patient's husband] was there, I knew he would tell me if he was going to go and lay down, or...go to get a meal...I knew I could just call out and he would be there for me...comforts me," *ARDS survivor number 339*.

The two key needs during this phase were the need for physical rehabilitation and the need for psychological counseling. This was especially important as the patients were transferred from the ICU to the general ward.

### Preparation for return to community living

This phase takes place just prior to patients returning home from either an acute care hospital or rehabilitation facility. This stage is characterized by a peak in information needs centered on discharge planning and available community resources. For example, this participant wanted to know and consider their care options:

"I would like to have had people talk to me a little bit more...about what to expect, like my choices, ...to go to a rehab home, go to a home, or stay a little longer," *ARDS survivor number 103*.

Participants also wanted to know what to expect and how to adapt to living at home. They wanted to know more about community resources and about how they should act or what they should or should not do to facilitate their recovery:

"...you've been in hospital,... primarily in bed for over a month, you are going to tire easily, you are not to ...stress your body, ...sometimes you need to be told. I'm used to not being sick...it just really never occurred to me that I couldn't just get up and split wood and do the whole nine yards," *ARDS survivor number 339*.

The uncertainty associated with returning to the community often left many patients feeling anxious and afraid about their discharge home. In contrast, many other patients were positive in their outlook and generally happy to be returning to a more familiar environment.

"I was really looking forward to being in my own home, because I definitely thrive better there...,"*ARDS survivor number 357*.

This variability in the emotional experiences of survivors was common in this phase and determined by the availability of supports at home. Patients who had no primary caregivers experienced more anxiety and fear, while those with family members and support networks were more optimistic and positive about their discharge.

Training and tangible needs in this phase focused predominantly on providing patients with information about accessing community care, vocational re-engagement, and social services. Survivors wanted to be able to continue with their recovery and rehabilitation, and return to doing every day activities once they were back in the community. As a result, participants wanted health care professionals to help facilitate this transition. For example, this survivor would have liked to receive more practical information and assistance to access these services:

"...my physiotherapist at [the rehabilitation centre] did research and found three physios for me, in the [home town] area, but that was pretty much all they did, they didn't give me any kind of information...like I couldn't drive, cause I couldn't walk... [husband] had to find a program for someone to come and pick me up and take me to physio....would be insane...I would say they did very, very, bare bones," *ARDS survivor number 352*.

In addition, at this time survivors were interested in relearning how to do daily activities including activities that will help them prepare to return to work.

"...extra training is good for me...I was a cook...after the accident...the occupational therapist...take me to my workplace...I forgot everything...chef you know...I had to relearn everything," *ARDS survivor number 424*.

### First few months of home adjustment

This phase is largely characterized by ARDS patients learning to re-integrate into independent community living. Dominant themes included the need for psychological counseling and rehabilitation services to improve recovery. Information needs still focused on their illness, but geared more towards coping with the long-term sequelae of their illness and stress. For example, this participant was not aware of the possible long-term sequelae so found it distressing when they started to experience some:

"See, I didn't know that was going to happen to me...like when they released me from the hospital I thought everything was perfectly fine...then slowly things kept creeping up on me....memory loss, trying to read and write,...trying to run a computer,...forgetting things about the kids...really crazy," *ARDS survivor number 118*.

During this early phase of home adjustment, many survivors discussed feeling emotionally unprepared for the transition home. Overall, the re-integration back into the community was a cause for increased stress and a source of depression. As a result, some patients felt they would like more support from community-based health care providers. Survivors also needed physical support at home and someone to help them through their emotional recovery.

"I guess in hindsight...I didn't feel like I had adequate support...I should have had someone coming in to give me a hand... I just felt scared and helpless,"*ARDS survivor number 339*.

"I definitely would have wanted [a counselor or therapist]...I'm a person that likes to spew. And I think if I would have had somebody to listen other then my husband, ...it would have been a real asset to me,"*ARDS survivor number 357*.

The tangible and training needs of patients follow in a similar fashion to their information needs. Family members continue to be a key source of support for the patients throughout the recovery process. Unfortunately, family support was not always perceived as being positive. This may be associated with the expectation that survivors should return to their previous level of functioning.

"I didn't understand...I thought she could be kind to me. She wasn't kind anymore. She saved my life, got me out of the hospital, brought me home, and then was mad and angry...maybe it was too much in retrospect to ask of my wife...my wife became my primary caregiver and that probably really ticked her off...she was mad at me....I was mad at her,"*ARDS survivor number 228*.

Support from peers became more important once the ARDS survivor returned to community living. Coping strategies represented a major component of the ARDS survivor's re-integration into the community and they looked to peers with similar experiences for guidance.

"if I had maybe a list of people or something, then, I might have considered calling...there might have been someone [ARDS survivor group] ...if I had questions I would have called just to have gotten some answers," *ARDS survivor number 345*.

#### Longer-term adjustment to community living

In this phase, the overriding concern was managing the long-term consequences of ARDS. Many of the support needs are geared towards secondary prevention of health events, ongoing health maintenance, and re-integration into society. Information needs persist into the adaptation phase and include not only information on the illness event, but also on monitoring, and prevention of future events. Survivors expressed a desire for information to be delivered in a more permanent fashion, either in the form of information pamphlets or booklets that could be reviewed again at their leisure. Survivors indicate a sense of wanting more information but not knowing where to obtain it.

"...I get the...articles [from the study] and I mean I just, I don't know where else to turn really. I don't know what to do to help myself,"*ARDS survivor number 363*.

Survivors also discussed the follow-up clinics that were part of the cohort study as a good source of information, specifically the physical testing. Information about the survivors current medical status seems to put survivors' and family members' minds at ease especially as they start to see the progress of their physical condition.

"...the study...you know in a way...helped me....I saw her [follow-up physician] twice a year, and...my wife feels more confident that....I was being looked at...and checked out...and the nice medical facilities here...have been a real plus...its just comforting going for pulmonary function...getting data and quantifying the situation," *ARDS survivor number 228*.

In this period survivors learn to manage the long-term consequences of ARDS. They continue to feel stress and anxiety over their future health, but happiness associated with their re-integration into the community was also common. Survivors continued to derive happiness from the support that they received from friends and family.

"I find that working and talking to my close girlfriends really help," *ARDS survivor number 419*.

"...my wife is here,...you know most of the time she advises me to do this or that...when I am angry or sad, I'm sitting here or sleeping, she said...you come with me, we go outside, trying to, always trying to make me feel better...," *ARDS survivor number 424*.

Instrumental needs are also present in this phase as survivors learn to "manage the long-term consequences of ARDS." In this phase, ARDS survivors foresee any future training or tangible needs that they may require in the years to come. Much of the needs focused on prevention, but some survivors also indicated the need for on-going care including clinical follow-up, rehabilitation, community care, and psychological counseling, as one survivor discusses:

"...I can see down the road where I may have to look at some kind of ...psychological counseling," *ARDS survivor number 339*.

Along with this, many survivors expressed an interest to give back to the community, and provide support to others who may experience the same illness.

"I'd like to go on to talk about this subject in a public sort of way, that would be helpful to others,"*ARDS survivor number 334*.

## Discussion

Our qualitative study used the TIR framework to identify changes in support needs as ARDS survivors moved across the care continuum. Although it was developed initially for the stroke population, the central premise - that support needs evolve over time - resonates with many patient populations including patients with critical illness. Using framework methodology allowed us to enter this qualitative study with this pre-existing idea, but also allowed us to explore the concept of changing experiences and support needs during the analysis. Our results indicate that the support needs of ARDS survivors did change across time and with expert clinical input we identified the key phases of recovery that ARDS survivors typically experience, and used these to guide our study. Throughout each of the five phases of recovery we identified informational, emotional, and instrumental needs. During the critical illness event and ICU care, survivors identified emotional comfort and knowledge transfer to family members as the most important features of this phase. Caregivers were identified as the primary source of information for patients once they left the ICU. The period of stabilization on the general ward was characterized by fear for their health and well-being because of decreased surveillance and the realization of the seriousness of their illness. As survivors were preparing for return to community living they wanted to ensure that appropriate resources were available to them in the community. During the first few months of home adjustment, ARDS survivors began to realize the long-term sequelae, which neither they nor their family members were prepared for. As a result, survivors felt they needed more support in the community, and more information to assist in their adjustment to living at home. During this phase, they were also interested in learning more from others who had a similar experience. After a longer-term adjustment to community living, survivors were concerned about the long-term consequences of ARDS, the prevention of future negative health events, and concern regarding return to work. They were unsure of where to find additional information, and wanted additional clinical care and psychological counseling. They appreciated any ongoing support provided by family members and friends.

In our study, early support needs were largely characterized by information needs surrounding their illness event, diagnosis, and prognosis. Family members played an important role in obtaining this information and sharing it with survivors when they moved to the general ward. In previous research, the use of diaries in fulfilling informational needs in the ICU has shown some positive impact on early post-ICU recovery [34]. This has further translated into a decrease in incidence of post-traumatic stress disorder in the survivors over the longer term [35]. Emotional needs were predominantly characterized by fear, frustration, and emotional distress. Many of these emotions stemmed from lack of familiarity in an environment and transitions to areas with less medical surveillance. Patients were unaccustomed to the sudden decrease in monitoring and assistance as they progressed through these early phases. These findings are not new, and in fact support findings seen in previous studies in coronary care unit patients [22,36]. More recently, Field and colleagues looked at the experiences of ICU patients and the stress associated with relocation to the general ward [37]. They noted that in addition to the physical and emotional difficulties relating to their illness and ICU care, communication, feeding, nursing care and support also contributed to this. [37].

Needs that occur outside of the ICU and general ward phases have not yet been articulated in the existing research. Our research provides guidance for future interventions because we consider a broad spectrum of needs as they occur across the recovery trajectory. Through our interviews we found a persistent need for information regarding the diagnosis and prognosis of ARDS throughout all the phases of recovery. This finding suggests that critical information needs that are not met early on in the recovery period persist throughout the care continuum until they are appropriately met. In the later phases of recovery, disease-specific information lessens and is replaced by needs surrounding resources on access to community care. Inconsistencies in the delivery of information were common suggesting that the delivery or transfer of knowledge from the health care team to the patients is variable. Emotional needs during the later phases were mixed. The anticipation of returning home brought with it challenges including difficulty coping with independent living, having unrealistic expectations for recovery, and coping with change in the relationships with their family caregivers. Re-integration back into the community ultimately determined the success of this phase. Instrumental needs during the later recovery phases focused on rehabilitation, vocational training, and access to care. Many survivors required ongoing reassurance about their health status and believed that in the future, there would be a need for more rehabilitation, psychological counseling, and medical follow-up. These qualitative findings are consistent with quantitative data of lower functional status and quality of life in ARDS survivors [6,8].

The results of this study are relevant and important because to date, no studies have looked at changing support needs in ARDS survivors using a recovery continuum such as the TIR framework.

The importance of family members and caregivers during the recovery period was therefore a major finding in this study. Family members acted as advocates for the survivors and this suggests that a family-centered approach towards care and recovery in the ICU would improve gaps in knowledge and care for the patients. Targeted interventions have been shown to improve functional performance and reduce hospital re-admissions and health care costs [38,39]. Only a few small studies have looked at interventions to aid families and caregivers during the recovery of a patient with critical illness [40-43]. These studies have largely helped to reduce anxiety and stress in the caregivers, and provide a source of communication between health care teams and family members of patients with critical illness [40-43]. A recent study by Prinjha and colleagues evaluated the perceptions of ICU patients on follow-up care [44]. In this study, patients felt that ICU follow-up services were important and contributed to their recovery in a positive way [44]. It also provided the patients with an opportunity to give feedback and receive information on their health status [44]. To date, only one randomized controlled trial looking at rehabilitation after critical illness has been performed [45]. In this study, the use of a self-help rehabilitation manual in addition to standard follow-up care in the UK found improvements in physical recovery and depression, but no difference in symptoms of anxiety or post-traumatic stress disorder. Recently, a randomized controlled trial looking at post-ICU discharge follow-up was completed in the UK [46]. Outcomes included health-related quality of life measures, neuropsychiatric measures, and health care utilizations. The results have not been formally published, but preliminary data appear to show no significant difference in these outcomes. It is possible that these interventions did not address the changing support needs appropriate for that period of recovery, and as a result, improvement in the study group was not seen. Future interventions may want to address these needs for maximal impact on outcomes.

Our study had some limitations. All participants in the study received care in a large urban centre, which may not be representative of all ICU survivors from smaller communities. The participants were part of a longitudinal cohort study where they received follow-up and clinical assessments, which currently are not standard of care. We suspect that if interviews were conducted during the phases of recovery, more support needs would be identified. In addition, some support needs (ie, information needs) may decrease over time because they are being identified and addressed early on. Most patients enrolled in this study were approximately six years post-ICU discharge. Recall bias is a potential problem as details of their experience may be lost, although it is likely that they are remembering their most significant experiences [47]. Lastly, member checking was not performed during this study to address whether our findings were representative of the survivors' experiences. However, three of the authors reviewed the transcripts and/or knew the participants well through clinical follow-up. As a result, we feel that the themes adequately represent the experiences of the participants as has been documented in the manuscript.

## Conclusions

This study has identified the changing needs of ARDS survivors as they progress from the ICU to their re-integration into community living. This study also provides a template for interventions to support survivors across their recovery trajectory. Presently, there is no system in place in the USA to provide organized follow-up for survivors of critical illness. In the UK, guidelines on 'Rehabilitation after critical illness' were published by the National Institute for Health and Clinical Excellence and provide consensus recommendations on the care of patients following a period of critical illness, and information on rehabilitation tools and support needs [27]. Future studies in this area would include a prospective needs assessment of ARDS survivors as they cross the care continuum to compare the results obtained from this study with those from a prospective study. Eventually, the goal would be to use the data obtained from our assessment to design an intervention program that meets the specific support needs of ARDS survivors across their recovery trajectory. This would then be validated using the outcomes from the original ARDS cohort. Other areas of interest would include creation of educational tools for both ARDS patients and their caregivers and potentially designing a multidisciplinary intervention to support the recovery of not only ARDS survivors, but also survivors of critical illness. Whether such a program would correspond to changes in quality of life and health outcomes following ARDS is still to be determined.

## Key messages

• The TIR framework provides structure for the development of a support program that encompasses the care continuum.

• ARDS patients have changing support needs throughout the recovery process.

• ARDS patients and their family caregivers have significant educational needs during and after the acute critical illness.

• Education and preparation throughout the course of recovery may decrease the negative health outcomes of ARDS.

## Abbreviations

ARDS: acute respiratory distress syndrome; FiO_2_: fraction of inspired oxygen; PaO_2_: partial pressure of arterial oxygen; TIR: timing it right.

## Competing interests

The authors declare that they have no competing interests.

## Authors' contributions

CML is the primary author that contributed to the design, acquisition of data, analysis and interpretation of data, as well as making significant contributions to the drafting and revising of the manuscript. MSH and JIC both made substantial contributions to the conception and design of the study, analysis and interpretation of data, and made revisions critically important to the intellectual content of the manuscript. AM contributed to the design and acquisition of data in the study. All authors were responsible for the final approval of the version of the manuscript to be published.
